# 

**Published:** 1893-12-30

**Authors:** 


**Notice to Advertisers and Subscribers.**

All Advertisements, Orders for Copies of The Hospital, and Business Communications should be addressed to The Publisher, *not to the Editor*, The Hospital, 428, Strand, W.C.

The Cost of Subscription, post free, is as follows:—Per week, 2½d.; per quarter, 2s. 6d.; per half-year, 5s.; per annum, 8s. 6d Foreign:—Per Annum, 10s. 8d.; payable, in all cases, in advance.

**OUR NEW OFFICES.**

428, Strand, W.C., will henceforth be the Office of this Journal.

**Notices of Births, Deaths, and Marriages** are inserted in this column at a charge of 2s. 6d. for an announcement not exceeding four lines.

DEATH.

Baker.—At Chalmers Hospital, Banff, on the 22nd inst., of typhoid fever, Mary Parkes Baker, eldest daughter of Thomas Baker, Esq., of Park View, Stirling, and Caledonian Tube Company, Coatbridge, dearly loved and deeply mourned.

### NOTICE TO CORRESPONDENTS.

All MS., Letters, Books for Review, and other matters intended for the Editor should be addressed The Editor, The Lodge, Porchester Square, London, W.

The Editor cannot undertake to return rejected MS., even when accompanied by stamped directed envelope.

**"Burdett's Hospital Annual," 1893.**

Fourth year of publication. 700 pp. Boards, gilt lettering. A most complete guide to British, Colonial, Indian, and American Hospitals, Dispensaries, Nursing and Convalescent Institutions, and Asylums. Price 5s. Published by The Scientific Press (Limited), 428, Strand, W.C.

**NOTICE.—The Index for Vol. XIV. (ending Sept. 30th, 1893) is on sale at the Office of this Journal, price 2½d., post free.**

### Scraps and Gleanings.

The total subscriptions received at the Plymouth Public Dispensary in the provident department during the last twelve months amounted to £387.

The Committee of the National Orthopædic Hospital have received a cheque for £5,436, the proceeds of a dance held at Queen's Gate Hall, on Wednesday, November 22nd.

The Ladies' Committee of the National Orthopædic Hospital held a sale of work on December 1 and 2, and realized over £98, to be devoted to the fund for aiding patients in the hospital.

A cafe chantant was recently organized at Southampton, which took place in the Philharmonic Hall, the proceeds being devoted to the funds of the Free Eye and Ear Hospital of that locality.

The Worshipful Company of Mercnant Taylors have sent a donation of a hundred guineas to the Central London Throat and Ear Hospital, Gray's-inn-road, towards the extension of that institution.

A sale of work was held early in November in aid of All Saints' Convalescent Home at St. Leonards. At the annual meeting of this institution the accounts were shown to leave a balance in hand of £11 to the good.

The ratepayers of the Isle of Thanet are protesting against the proposed acquisition of the "Brickfield" estate as a site for the New Isle of Thanet, Urban, Joint Hospital. They are urging for the purchase of another site of larger proportions, and at a lower figure.

The Crystal Palace Company are now urging substantial reasons against the erection of a fever hospital at the Grangewood Estate, Upper Norwood. This project, if continued in, will involve serious pecuniary losses to this recreation ground of over 2,500,000 annual visitors.

An excellent scheme is afloat at Leamington. It has been proposed to establish a convalescent home for children at Kenilworth, near the Common, where delicate Warwickshire children, between five and eight years of age, requiring change of air can be supplied with a fortnight's rest and care.

At the annual meeting of the governors of the Longton Cottage Hospital, the report for the year presented a more satisfactory financial condition than had been anticipated, receipts from all sources on the hospital accounts having amounted to £949 during the last twelve months.

The committee which was appointed at the last annual meeting of the Northampton General Infirmary to consider the question of a new operating-room for the institution, have now presented a report and plans showing a large operating-room and new surgery at a probable cost of £2,500

The report for the St. Mary's Cottage Hospital at Southampton has just been issued, from which we gather that during the twelve months under review 448 patients were admitted for treatment. The private subscription ball held in Piccadilly realised £210 in favour of the funds of the institution.

The committee of the Reigate and Redhill Cottage Hospital in presenting the 27th annual report remarked that the usefulness of the institution was well maintained. Subscriptions and donations, however, show a falling off, and an appeal in favour of the hospital funds will shortly be started.

The sum of £280 has been paid to the treasurer of the Reading Dispensary by the trustees of the late Volunteer Fire Brigade of that town.

The annual meeting of the Carnarvonshire and Anglesey Infirmary was held lately. The financial report showed a balance of £7,476 in the treasurer's hands.

A large public meeting was held recently at Streatham, at which Mr. W. F. Penfold was presented with an illuminated address and gold chronometer and chain in recognition of his active endeavours for the preservation of Tooting Bec Common from the disadvantages of a proposed fever hospital.

The first general meeting of the promoters of the National Hospital for Consumption for Ireland was recently held. The Marchioness of Zetland, whose exertions on behalf of the proposed institution have been so valuable, has allowed her name to stand as first patroness of the association.

The grand bazaar at Salisbury, which was held last month, was a great success. It was formally opened by Lady Radnor, and was well attended during the three days it remained open. The proceeds amounted to £1,368, exclusive of extensive collections made for the funds of the hospital in the villages round Salisbury.

The Board of the Ventnor Hospital for Consumption are lamenting the heavy deficit which exists on the funds of the institution at this time of the year. The diet required for the patients is nourishing and expensive, and the hospital, being worked on the separate system, adds two very serious items to the expenses of its maintenance.

As is pretty generally known, the accommodation at the Infectious Diseases Hospital at Winson Green, Birmingham, has long since been overtaxed by the scarlet fever outbreak. With the possibility of a prolonged epidemic of small-pox, the Health Committee have taken the step of buying a piece of land at Yardley for the erection of a fever hospital.

The Board of Management of the Chelsea Hospital for Women have done a graceful and kindly act in placing at the disposal of the Actors' Benevolent Fund for the ensuing year, a bed in the hospital at Chelsea, and one in the Convalescent Home at St. Leonards-on-Sea, "for the benefit of those less fortunate members of the dramatic profession, who may be laid aside by sickness and suffering."

A complimentary dinner was given by the Council and medical staff of the Royal Chest Hospital, City Road, at the Albion Hotel, Aldersgate Street, E.C., on Saturday, December 9th, to Mr. Hope Morley, the treasurer, and Mr. T. Andros de la Rue, the Chairman of the Council of Management. The Right Hon. the Lord Charles Bruce, the President of the hospital, was in the chair, and among the guests were: The Rev. W. G. Abbott, M.A., Mr. George Berry, Colonel W. B. Birch, the Rev. J. H. Rose, M.A., Mr. Gilbert Smith, Dr. W. H. White, Mr. Willoughby S. Smith, Mr. Charles J. Webb, Dr. James Calvert, Dr. Arthur Davies, Dr. Schorstein, Dr. Bowman, Dr. Dawson, and Mr John Harrold, the secretary of the hospital..

### Books Received.

Hay Nisbet and Co.

"Hints on Home Nursing." By Dr. R. Halliday.

Cassell and Co.

"Diseases of the Skin." By Malcolm Morris.

**Periodicals and Pamphlets.**—Child's Guardian, London. The Sun, Melbourne. Amateur Gardening. Sylvia's Home Journal (Christmas number). The Homœopathic Review. Public Health. Cassell's Family Magazine. Boy's Own Paper. Girl's Own Paper. Leisure Hour. Sunday at Home. The Young Gentlewoman. The Butterfly. The Sun, London. Best 6d. Cookery. Guy's Hospital Gazette. Spinal Curvature. Nursing Notes (Vol. vi.). The Medical Pioneer. The Land Roll.

### The Hospital Advertiser.

### MEDICAL VACANCIES AND APPOINTMENTS.

**APPOINTMENTS.**

E. Baines, B.A.. M.B., B.C., of Leeds, to the post of House Surgeon of the Sunderland Infirmary. W. P. Blumer, F.R.C.S. Edin., to the post of Honorary Surgeon to the Sunderland Infirmary. C. C. Burt, appointed Medical Officer for the Snitterfield District of the Stratford-on-Avon Union. J. F. Carruthers, M.B., C.M.Edin., appointed Medical Officer for the Tattershall District of the Horncastle Union. T. W. Cole, B.A.Dub.. M.B., appointed Medical Officer of Health to the Bolsover Local Board. W. M. Cox, M.R.C.S., L.R.C.P., appointed Resident Medical Officer to the Birmingham and Midland Free Hospital for Sick Children. A. Crawford, appointed Second Assistant Medical Officer of the Infirmary of the parish of St. Leonard, Shoreditch. T. Crowther, M.D., M.R.C.S., appointed Joint Medical Officer of Health for Warley, Luddenden Foot, and Midgley. W. R. Etches, M.R.C.S., L.R.C.P., D.P.H.Lond., appointed Medical Officer of Health for Macclesfield Urban Sanitary Authority. W. W. Jones, M.B., M.Ch.Edin., D.P.H.Camb., appointed Medical Officer to the South Wales and Monmouthshire Truants' School. W. R. Jordan, M.B.Lond., appointed Extra Acting Physician to the Birmingham and Midland Free Hospital for Sick Children. J. V. Laverick, L.R.C.P.Lond., L.F.P.S.Glasg., appointed Medical Officer of Health to the Whitby Rural Sanitary District. J. B. Lendrum, M.B., M.S.Aberd., appointed Assistant House Surgeon to the Huddersfield Infirmary. H. Lockwood, M.R.C.S.Eng., L.S.A., appointed Honorary Surgeon to the Sheffield Public Hospital. J. W. Mactavish, appointed Medical Officer of the Lowdham District of the Southwell Union. T. W. S. Morgan, M.R.C.S.Eng., appointed Medical Officer for the Eighth District of the Bedminster Union. A. W. Sheperd, L.R.C.P., L.R.C.S.I., appointed Medical Officer of Health to the Cowbridge Town Council. W. B. Smith, M.R.C.S., L.R.C.P., appointed District Medical Officer to the Salford Royal Hospital. W. B. Stanford, appointed Assistant Medical Officer to the Islington Workhouse Infirmary. C. A. Thorne, L.R.C.P., L.R.C.S.Edin., appointed Medical Officer of the Sixth District of the Ecclesall Bierlow Union. G. E. Knight Thorpe, M.R.C.S.Eng., appointed Honorary Consulting Surgeon to the Sheffield Public Hospital. C. Byron Turner, M.R.C.S.Eng., L.R.C.P.Lond., appointed Assistant Surgeon to the Grimsby and District Hospital. C. S. Wallace, F.R.C.S., L.R.C.P., appointed House Surgeon to St. Thomas's Hospital (extension). S. W. C. Warneford, M.R.C.S., L.R.C.P.Lond., appointed Medical Officer of the Harbury District of the Southam Union. Neish Park Watt, M.A., M.B., C.M.Edin., appointed House Surgeon to Gray's Hospital, Elgin. A. Westwood, appointed Medical Officer for the Stretford District of the Barton-upon-Irwell Union. J. A. T. White, M.R.C.S., L.R.C.P., appointed House Physician to the Metropolitan Hospital, Kingsland Road, N. Edwin St. John Whitehouse, M.R.C.S., L.R.C.P., appointed Resident Surgical Officer to the Birmingham and Midland Free Hospital for Sick Children. A. H. Woodcock, L.R.C.P., M.R.C.S., appointed Clinical Assistant in the Department for Diseases of the Ear, St. Thomas's Hospital (extension). Herbert Hunter Woods, L.R.C.P.Lond., M.R.C.S.Eng., appointed House Physician to Charing Cross Hospital. Meredith Young, M.B., C.M.Edin., Medical Officer of Health Borough of Brighouse, appointed Medical Officer of Health to the Rural Sanitary District of the Halifax Union.

**VACANCIES.**

The following vacancies are announced this week, the amount of salary in each case being given after the name of the institution:

**Resident Medical Officers.**

Bristol City Lunatic Asylum. Second Assistant Medical Officer, duly qualified in medicine and surgery. Salary £120 per annum, with furnished apartments, board, and washing. Applications and testimonials to the Medical Superintendent, at the Asylum, Fishponds, near Bristol, by January 5th, 1894.

Central London Ophthalmic Hospital, Gray's Inn Road, W.C. House Surgeon. Applications, with testimonials, to the Secretary before January 8th, 1894.

Horton Infirmary, Banbury. House Surgeon and Dispenser, duly qualified and registered. Salary £60 per annum, with board and lodging. Applications and testimonials to C. H. Davids, 21, Marlborough Road, Banbury, by January 6th, 1894.

Royal London Ophthalmic Hospital, Moorfields, E.C. Junior House-Surgeon. Salary £50 per annum, with board and residence. Applications and testimonials to be sent to the Secretary by January 4th, 1894.

Victoria Hospital for Sick Children, Queen's Road, Chelsea, S.W. House Surgeon and House Physician to the In-patients. Honorarium £50 each per annum, with board and lodging in the hospital. Applications to the Secretary by January 13th, 1894.

### Non-Resident Medical Officers.

Dental Hospital of London, Leicester Square. Dental Surgeon; must be a Licentiate in Dental Surgery. Applications to J. Francis Pink, Secretary, by January 8th, 1894.

Surrey Dispensary, Great Dover Street, S.E. Surgeon. Honorarium £52 10s. per annum. Applications to J. Harrison, 179, Bermondsey Street, S.E., before January 9th, 1894.

---

### FOOTBALL.

**Westminster Hospital v. Dental Hospital.—Played on Athletic Grounds, Kensal Rise, won by the former club by 4 goals and 5 tries to *nil*. During the second half one of the Westminster team, R. Nitch-Smith, had the misfortune to break his right fibula.**

---

### PASS LISTS.

### University of London.

M.D. EXAMINATION, 1893.

Medicine.—C. Addison, B.S., St. Bartholomew's Hospital; W. L. Andriezen, University College; H. A. Ballance, B.S., University College; S. H. Bates, University College; Annette M. Benson, B.Sc., London School of Medicine and Royal Free Hospital; G. F. Blacker,* B.S., University College; A. S. Blackwell, B.S., B.Sc., St. Bartholomew's Hospital; W. Bligh, B.S., Guy's Hospital; C. R. Box, B.S., B.Sc., St. Thomas's Hospital; A. N. Boycott, St. Thomas's Hospital; T. G. Brodie, King's College; S. Bueno de Mesquita, Guy's Hospital; H. A. Burrowes, Royal Infirmary, Liverpool; F. J. Coleman, B.S., Guy's Hospital; J. N. Collins, London Hospital; H. G. G. Cook, B.S. (gold medal), St. Bartholomew's Hospital; C. W. Cooke, St. Thomas's Hospital; T. P. Cowen,* B.S., St. Thomas's Hospital; A. M. Daldy, B.S., Guy's Hospital; T. B. P. Davies, M.S., Guy's Hospital; B. E. Dawson, B.Sc., London Hospi al; H. A. Eccles, St. Bartholomew's Hospital; C. C. Elliott, B.S., Guy's Hospital; H. M. Evans, University College; J. Evans, University College, Liverpool; H. Finley, University College; E. T. E. Hamilton, B.S., B.Sc., Guy's Hospital; A. D. Heath, University College; J. Jones, B.S., University College; H. E. Knight, St. Bartholomew's Hospital; H. Langdale, Owens College and Manchester Royal Infirmary; Alice J. McLaren, B.S., London School of Medicine and Royal Free Hospital; C. B. T. Musgrave, University College; H. T. Parker, B.S., St. Bartholomew's Hospital and St. Jacob's Krankenhaus, Leipzig; W. L. Pethybridge, B.Sc., St. Bartholomew's Hospital; R. Pickard, M.S., St. Bartholomew's Hospital; W. J. Pickup, University College; C. H. Preston, B.S., Owens College and Manchester Royal Infirmary; E. L. Pritchard, King's College; W. G. Rogers, M.S., Guy's Hospital; C. E. Salter, B.S., Guy's Hospital; H. S. Sandifer, B.S., King's College; A. W. Sheen, B.S., Guy's Hospital; H. R. Smith, University College; R. V. Solly, B.S., St. Thomas's Hospital; G. A. Stephens, B.S., B.Sc., University College; W. H. Sturge, London Hospital; H. Symons, St. Bartholomew's Hospital; W. W. H. Tate, University College; A. E. Tebb, B.S., Guy's Hospital; F. W. Tunnicliffe, St. Bartholomew's Hospital; E. E. Ware, B.S., St. Thomas's Hospital; F. W. Wesley, B.S., University College; C. E. Wheeler, B.S., B.Sc., St. Bartholomew's Hospital and St. Jacob's Krankenhaus, Leipzig; A. Whitfield, King's College.

State Medicine.—S. Barwise, Queen's and Mason Colleges, Birmingham; G. S. Buchanan, M.D., B.S., B.Sc., St. Bartholomew's Hospital and University College; H. M. Richards, M.D., B.S. (gold medal), University College; H. Williams, M.D., St. Bartholomew's Hospital.

M.S. EXAMINATION, 1893.

F. C. Abbott,* B.Sc., St. Thomas's Hospital; T. J. Dyall, M.D., St. Bartholomew's Hospital; J. L. Firth, M.D., University College; A. D. Fripp, Guy's Hospital; B. G. A. Moynihan (gold medal), Leeds School of Medicine; H. J. Waring,* B.Sc., St. Bartholomew's Hospital.

* Obtained the number of marks qualifying for the gold medal.

B.S. EXAMINATION, 1893.

First Division.—Louisa B. Aldrich-Blake, London School of Medicine and Royal Free Hospital; H. W. Armstead, St. Bartholomew's Hospital; J. Le M. Bunch, B.Sc., University College; T. Carwardine, Middlesex Hospital and University College; F. P. S. Cresswell, B.Sc., Guy's Hospital; H. O. Davies, St Bartholomew's Hospital; P. S. Eves, University College; C. S. Jaffé, St. Thomas's Hospital; W. J. Johnson, Guy's Hospital; W. H. Kelson, M.D., London Hospital; V. W. Low, St. Mary's Hospital; A. Manknell, Yorkshire College; J. A. Pickels, Owens College and Manchester Royal Infirmary; A. L. Whitehead, Yorkshire College.

Second Division.—V. B. Bennett, University College, Liverpool; J. G. Clegg, Owens College and Manchester Royal Infirmary; J. H. Griffiths, St. Bartholomew's Hospital; F. Hazell, Guy's Hospital; T. H. Ionides, University College; E. A. Nathan, St. Mary's Hospital; A. Paine, St. Mary's Hospital; W. P. Purvis, B.Sc., St. Thomas's Hospital; E. J. Smyth, B.Sc., University College; F. T. Travers, University College; W L. Wainwright, St. Thomas's Hospital.

